# Supplementary material for: Depletion of Embryonic Macrophages Leads to a Reduction in Angiogenesis in the Ex Ovo Chick Chorioallantoic Membrane Assay
Source: Cells. 2020 Dec 22;10(1):5. doi: 10.3390/cells10010005 (PMC7822194; doi:10.3390/cells10010005)
Supplement: Supplementary file 1 [file cells-10-00005-s001.pdf]

Supplementary Information

Title

Depletion of embryonic macrophages leads to a reduction in angiogenesis in the ex ovo chick chorioallantoic membrane assay

Authors

Hanna Tay[1,2], Charis Du Cheyne[1], Kristel Demeyere[3], Jurgen De Craene[1], Lobke De Bels[1], Evelyne Meyer[3], Andries Zijlstra[4], Ward De Spiegelaere[1,2]

Author affiliations

[1] Department of Morphology, Ghent University, Merelbeke, East-Flanders, Belgium

[2] Cancer Research Institute Ghent (CRIG), Ghent University, Merelbeke, East-Flanders, Belgium

[3] Department of Pharmacology, Toxicology and Biochemistry, Ghent University, Merelbeke, East-Flanders, Belgium

[4] Department of Pathology, Microbiology and Immunology, Vanderbilt University Medical Center, Nashville, Tennessee, USA

The supplementary information includes 7 figures and 3 tables

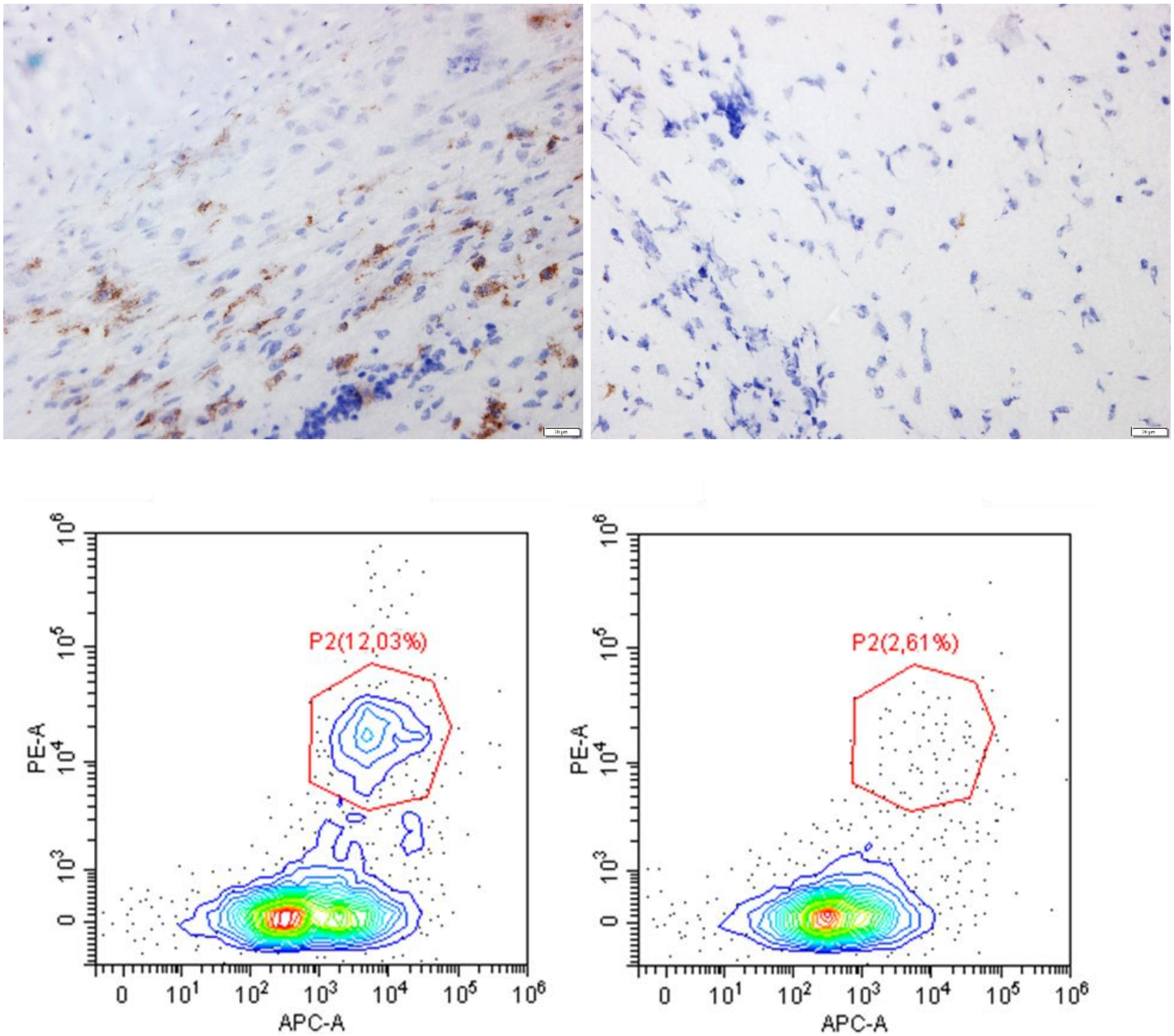

Supplementary Figure 1 The depletive effect on chicken macrophages after injection of 100 or 50 µl of clodronate liposomes (right image and density plot) versus 100 or 50 µl of PBS liposomes (left image and density plot) is comparable to that after the injection of 25 µl of liposomes. The immunohistochemistry images are an example of the decrease in KUL01-positive stained macrophages after injection of 100 µl clodronate liposomes, while the density dot-plots show the reduction of PE-KUL01 stained macrophages in the CD45-APC stained leukocyte population after injection of 50 µl of clodronate liposomes.

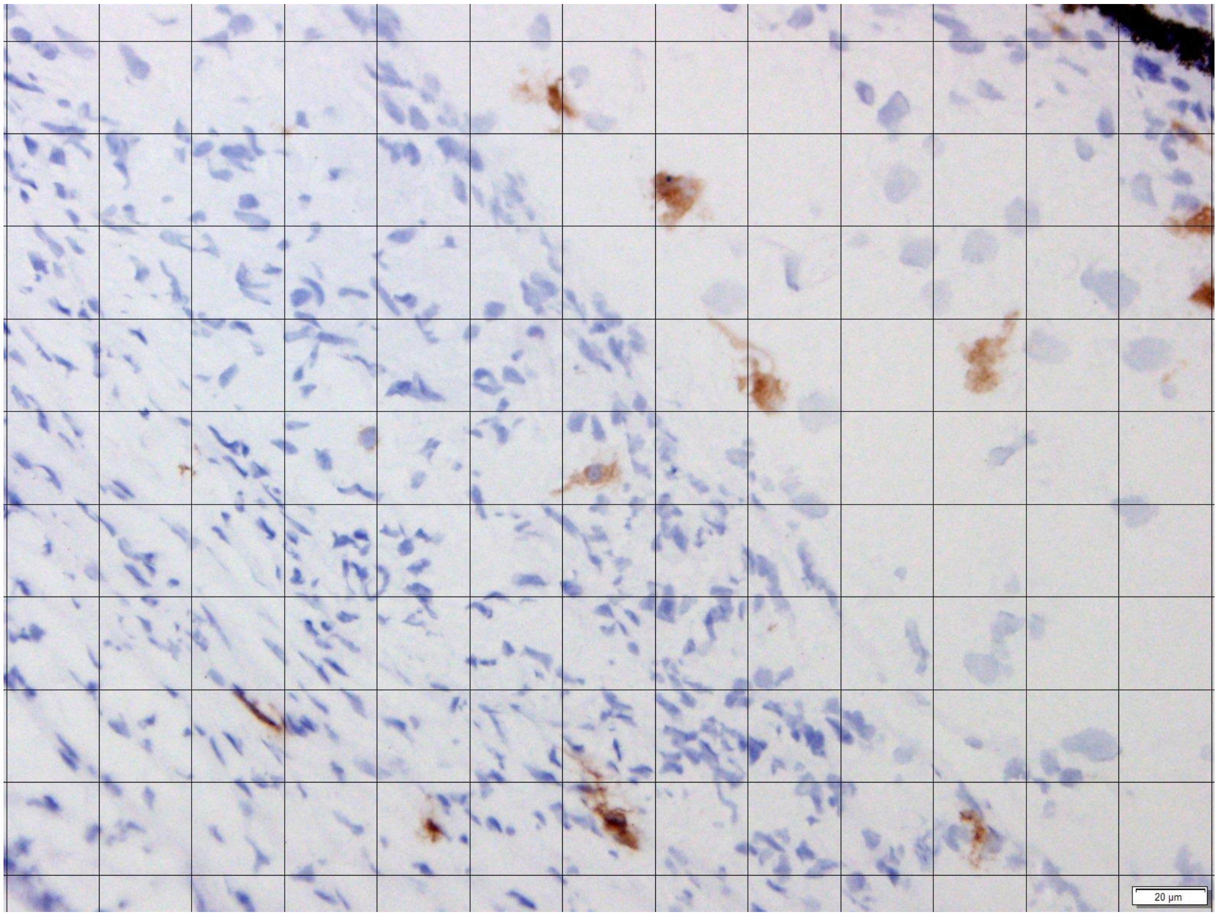

Supplementary Figure 2 Random placement of a grid on immunohistochemistry images allowed for the quantification of macrophages. The amount of positive cells and number of crossing points of the grid with embryonic tissue was determined. Then, the ratio of positive cells per crossing point was calculated.

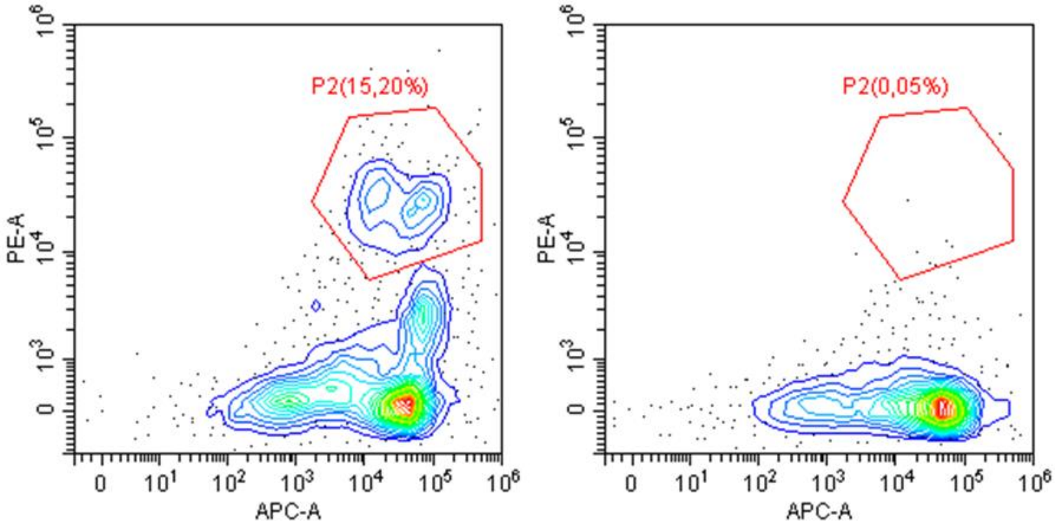

Supplementary Figure 3 Staining with an isotype control (right) shows that the background signal is negligible and that the observed PE-signal was primarily due to the specific binding of the PE-labelled KUL01 antibody to chicken macrophages (left).

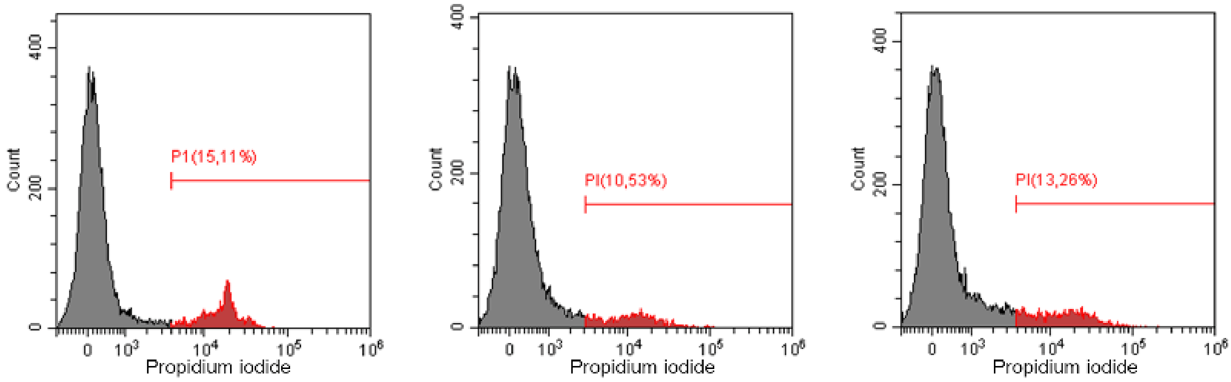

Supplementary Figure 4 Cell viability after sample preparation for flow cytometry varied between 85-90% (biological triplicates). This % viability is calculated as 100 - % PI^+. PI = propidium iodide.

Supplementary Table 1 Overview of the embryo survival rate in each CAM angiogenesis experiment.

Experiment	Treatment	Embryos injected	Embryos survived	Survival (%)
1	PBS liposomes	8	7	87.50
	Clodronate liposomes	9	4	44.44
2	PBS liposomes	8	6	75.00
	Clodronate liposomes	9	4	44.44
3	PBS liposomes	8	7	87.50
	Clodronate liposomes	9	5	55.56
4	PBS liposomes	8	7	87.50
	Clodronate liposomes	9	5	55.56
5	PBS liposomes	8	6	75.00
	Clodronate liposomes	10	8	80.00
6	PBS	7	6	85.71
	Free clodronate	8	7	87.50
7	PBS	7	7	100
	Free clodronate	8	7	87.50
8	PBS	5	4	80.00
	Free clodronate	6	4	66.67

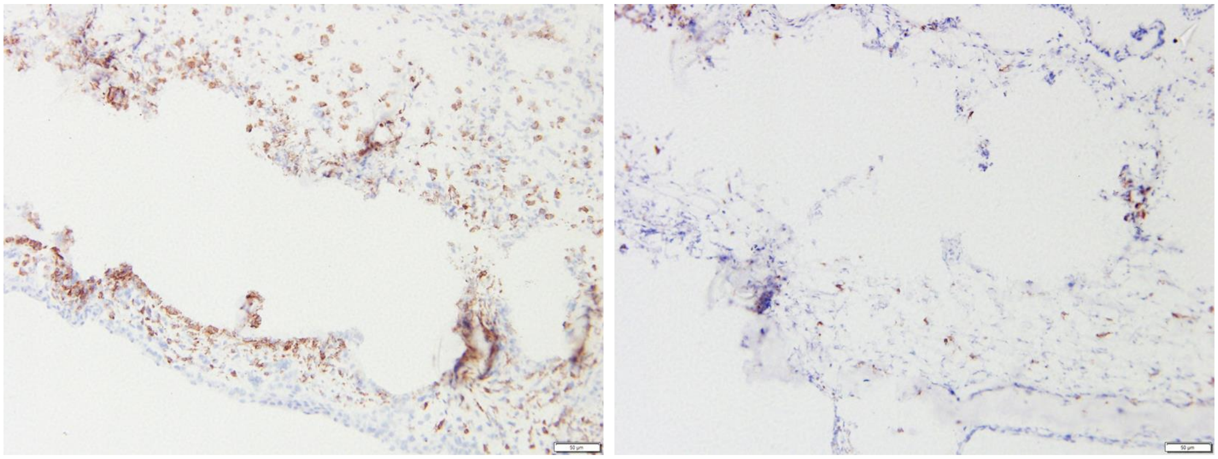

Supplementary Figure 5 Collagen/mesh onplants were recuperated for immunohistochemistry in the same fashion as the embryonic samples. During cutting of the collagen/mesh onplants, the samples easily ripped at the site where the nylon mesh was located. However, the rip and thus the area where the mesh had been, could easily be detected. In control embryos, a large amount of KUL01 positive macrophages could be detected in the CAM surrounding the area where the mesh was previously located (left). In clodronate liposome-injected embryos, the number of positive cells was considerably lower (right), confirming that depletion of macrophages was also detected at the level of the collagen/mesh onplants.

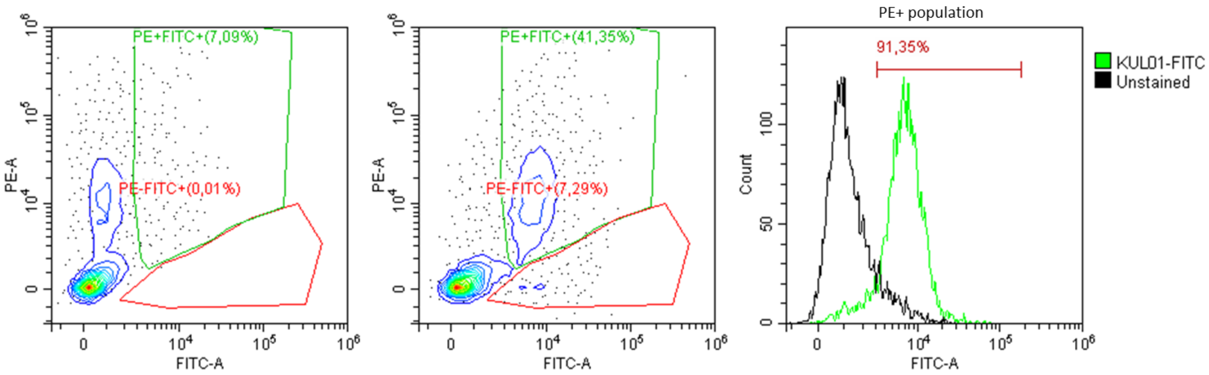

Supplementary Figure 6 Injection of fluorescent (PE) Dil-liposomes and subsequent analysis with flow cytometry confirmed that these PE-positive liposomes are mainly ingested by chicken macrophages (detectable with KUL01-FITC conjugated antibody). The two density- dot plots (unstained - (left panel) and stained with (middle panel) KUL01-FITC) show that the PE-positive subpopulation, which represents the cells that have ingested Dil-liposomes, shifts towards the right after staining with KUL01-FITC, indicating that the majority of liposomes is ingested by macrophages. The PE-negative KUL01-FITC-positive subpopulation depicts the macrophages that have not ingested Dil-liposomes (7-8% of the leukocyte population).The histogram (right panel) shows the FITC signal after gating of all PE (Dil-liposomes)-positive events either unstained (black) or stained with (green) KUL01-FITC conjugated macrophage marker The peak shifts to the right when the Dil-liposome ingesting population is stained with KUL01-FITC indicating that 91.35 % of these cells are KUL01-FITC positive.

Supplementary Table 2 Overview of the embryo survival rate in each CAM angiogenesis experiment.

Experiment	Treatment	Embryos injected	Embryos survived	Survival (%)
1	PBS liposomes	8	7	87.50
	Clodronate liposomes	9	4	44.44
2	PBS liposomes	8	6	75.00
	Clodronate liposomes	9	4	44.44
3	PBS liposomes	8	7	87.50
	Clodronate liposomes	9	5	55.56
4	PBS liposomes	8	7	87.50
	Clodronate liposomes	9	5	55.56
5	PBS liposomes	8	6	75.00
	Clodronate liposomes	10	8	80.00
6	PBS	7	6	85.71
	Free clodronate	8	7	87.50
7	PBS	7	7	100
	Free clodronate	8	7	87.50
8	PBS	5	4	80.00
	Free clodronate	6	4	66.67

Supplementary Table 3 Overview of the angiogenic response (percentage) in all collagen plugs used in the CAM angiogenesis assay

Experiment	Embryo	Treatment	Onplant treatment	Percentage
1	1	PBS liposomes	Control	0,00
			Control	0,00
			HT1080	14,58
			HT1080	29,14
	2	PBS liposomes	Control	4,69
			HT1080	47,00
			HT1080	23,61
	3	Clodronate liposomes	Control	0,00
			Control	0,00
			HT1080	0,00
	4	PBS liposomes	Control	10,26
			Control	10,39
			HT1080	55,56
			HT1080	5,56
	5	Clodronate liposomes	Control	0,00
			Control	3,57
			HT1080	1,85
			HT1080	0,00
	6	Clodronate liposomes	Control	0,00
			Control	0,00
			HT1080	4,55
			HT1080	11,90
			HT1080	5,00
	7	PBS liposomes	Control	0,00
			Control	0,00
			HT1080	0,91
			HT1080	0,00
	8	Clodronate liposomes	Control	2,44
			Control	0,00
			HT1080	2,78
			HT1080	1,59
	9	PBS liposomes	Control	0,00
			Control	1,67
			HT1080	2,60
			HT1080	2,48
	10	PBS liposomes	Control	1,59
			HT1080	8,33
2	11	PBS liposomes	Control	50,67
			Control	64,81
			HT1080	87,50
	12	Clodronate liposomes	Control	0,00
			Control	0,00
			HT1080	0,00
			HT1080	0,00
	13	PBS liposomes	Control	5,00
			Control	1,79
			HT1080	10,29
			HT1080	10,94
	14	Clodronate liposomes	Control	2,22
			Control	0,00
			HT1080	11,57
	15	PBS liposomes	Control	8,40
			Control	2,27
			HT1080	13,41
			HT1080	14,53
	16	Clodronate liposomes	Control	0,00
			Control	1,18
			HT1080	10,71
			HT1080	16,16
	17	PBS liposomes	Control	10,58
			Control	5,77
			HT1080	60,71
			HT1080	42,86
	18	Clodronate liposomes	Control	0,00
			Control	48,61
			HT1080	3,13
			HT1080	0,00
	19	PBS liposomes	Control	7,41
			Control	1,67
			HT1080	0,00
	20		Control	0,00

		Clodronate liposomes	Control	0,00
			HT1080	0,00
			HT1080	0,00
	21	PBS liposomes	Control	55,56
			Control	7,95
			HT1080	8,65
			HT1080	1,18
3	22	PBS liposomes	Control	0,83
			Control	7,14
			HT1080	0,57
			HT1080	13,33
	23	Clodronate liposomes	Control	7,14
			Control	3,33
			HT1080	68,75
			HT1080	0,00
	24	PBS liposomes	Control	14,00
			Control	4,17
			HT1080	13,10
			HT1080	1,35
	25	Clodronate liposomes	Control	0,00
			Control	0,00
	26	PBS liposomes	Control	16,78
			Control	10,71
			HT1080	34,29
			HT1080	63,64
	27	Clodronate liposomes	Control	8,93
			HT1080	8,00
	28	PBS liposomes	Control	8,33
			Control	4,44
			HT1080	28,67
			HT1080	10,77
	29	Clodronate liposomes	Control	0,00
			Control	0,00
			HT1080	5,13
			HT1080	8,33
	30	PBS liposomes	Control	2,27
			Control	1,30
			HT1080	5,36
	31	Clodronate liposomes	Control	0,00
			HT1080	0,00
	32	PBS liposomes	Control	0,00
			Control	0,00
			HT1080	6,92
			HT1080	0,00
	33	PBS liposomes	Control	3,57
			Control	21,68
			HT1080	23,81
			HT1080	44,32
4	34	PBS liposomes	Control	4,55
			Control	6,49
			HT1080	19,70
			HT1080	0,00
	35	Clodronate liposomes	Control	0,00
			Control	9,26
			HT1080	0,00
			HT1080	10,71
	36	PBS liposomes	Control	9,38
			Control	14,06
			HT1080	13,75
	37	Clodronate liposomes	Control	1,00
			Control	0,00
			HT1080	0,00
			HT1080	2,86
	38	PBS liposomes	Control	2,60
			Control	1,92
			HT1080	6,78
			HT1080	6,92
	39	Clodronate liposomes	Control	0,00
			Control	0,00
			HT1080	1,12
			HT1080	0,00
	40	PBS liposomes	Control	0,00
			Control	0,00
			HT1080	0,00
			HT1080	0,00
	41	PBS liposomes	Control	13,59
			Control	2,86

			HT1080	47,25
			HT1080	35,37
	42	Clodronate liposomes	Control	7,94
			HT1080	5,56
	43	PBS liposomes	Control	1,65
			Control	12,34
			HT1080	23,08
			HT1080	9,52
	44	PBS liposomes	Control	12,00
			Control	4,76
			HT1080	19,15
			HT1080	25,76
5	45	Clodronate liposomes	Control	0,00
			Control	1,01
			HT1080	5,30
			HT1080	11,11
	46	PBS liposomes	Control	6,94
			Control	2,20
			HT1080	33,68
			HT1080	0,00
	47	Clodronate liposomes	Control	0,00
			Control	7,19
			HT1080	16,67
			HT1080	23,81
	48	PBS liposomes	Control	12,99
			Control	3,79
			HT1080	8,97
			HT1080	69,44
	49	Clodronate liposomes	Control	10,00
			Control	3,33
			HT1080	2,02
			HT1080	14,88
	50	Clodronate liposomes	Control	1,79
			Control	3,33
			HT1080	0,56
			HT1080	18,18
	51	PBS liposomes	Control	1,67
			Control	22,73
			HT1080	17,78
			HT1080	47,44
	52	Clodronate liposomes	Control	0,00
			Control	6,25
			HT1080	12,22
			HT1080	8,33
	53	PBS liposomes	Control	8,33
			HT1080	30,61
	54	Clodronate liposomes	Control	3,53
			Control	0,00
			HT1080	3,33
			HT1080	1,39
	55	PBS liposomes	Control	1,67
			Control	1,67
			HT1080	38,89
			HT1080	10,00
	56	Clodronate liposomes	Control	12,12
			Control	1,79
			HT1080	25,48
			HT1080	4,90
	57	PBS liposomes	Control	9,40
			Control	12,86
			HT1080	3,90
			HT1080	8,33
	58	Clodronate liposomes	Control	20,00
			Control	4,76
			HT1080	0,83
			HT1080	27,08

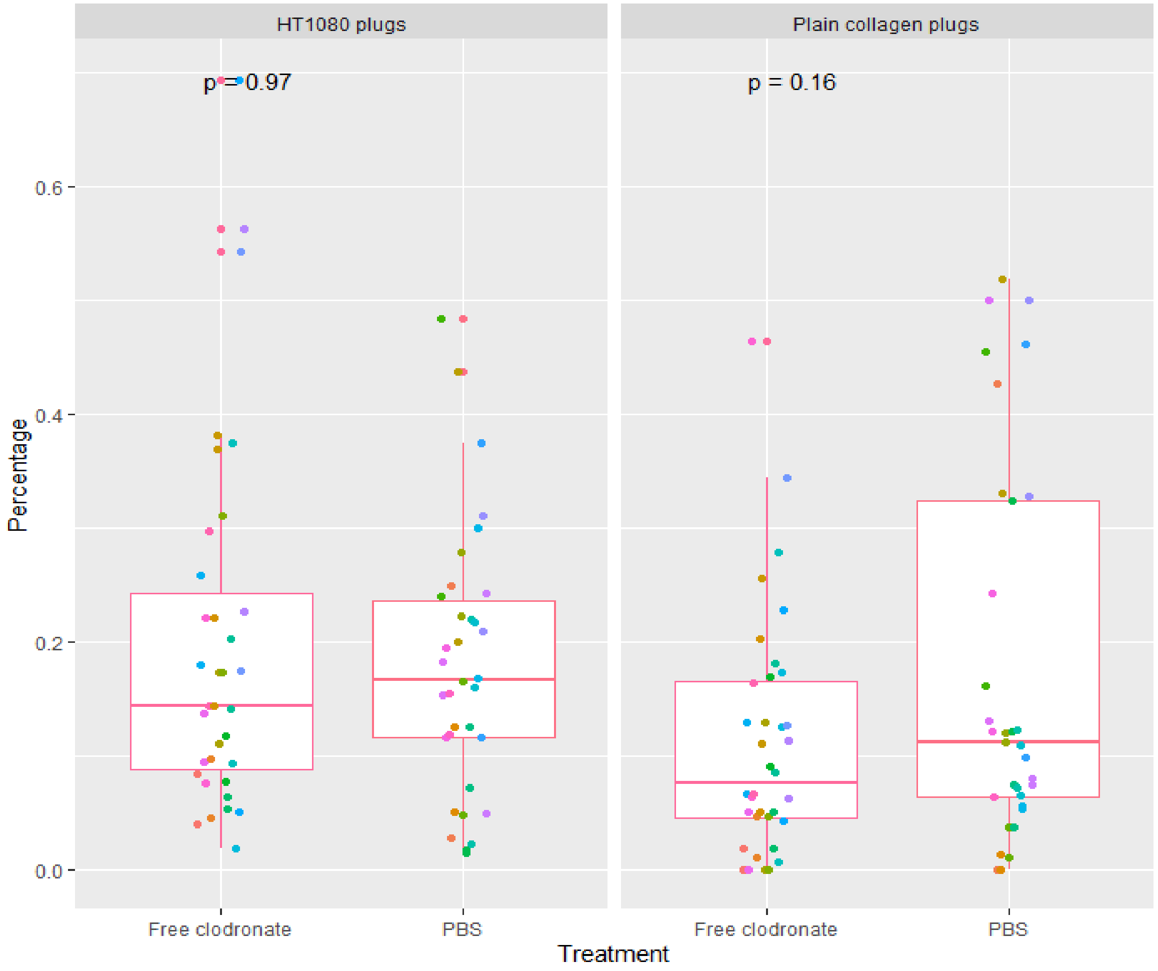

Supplementary Figure 7 Injection with free clodronate did not have a significant effect on angiogenesis compared to injection with PBS. Statistical analysis using the Wilcoxon signed-rank test revealed that no significant difference could be observed in the amount of ingrowing capillaries in HT1080 plugs (P = 0.97) as well as in plain collagen plugs (P = 0.16). Data are presented as boxplots showing the median, first and third quartiles.
